# A Novel Method of Mouse RPE Explant Culture and Effective Introduction of Transgenes Using Adenoviral Transduction for *In Vitro* Studies in AMD

**DOI:** 10.3390/ijms222111979

**Published:** 2021-11-05

**Authors:** Peng Shang, Nadezda A. Stepicheva, Haitao Liu, Olivia Chowdhury, Jonathan Franks, Ming Sun, Stacey Hose, Sayan Ghosh, Meysam Yazdankhah, Anastasia Strizhakova, Donna Beer Stolz, J. Samuel Zigler, Debasish Sinha

**Affiliations:** 1 Department of Ophthalmology, Children’s Hospital of University of Pittsburgh School of Medicine, One Children’s Hospital Drive, 4401 Penn Avenue, Pittsburgh, PA 15224, USA; nstepich@pitt.edu (N.A.S.); hal140@pitt.edu (H.L.); CHOWDHUR@pitt.edu (O.C.); stacey.hose@pitt.edu (S.H.); sayang@pitt.edu (S.G.); meysam_yazdankhah@yahoo.com (M.Y.); strizhak@pitt.edu (A.S.); Debasish@pitt.edu (D.S.); 2 Department of Cell Biology and Center for Biologic Imaging, University of Pittsburgh School of Medicine, Pittsburgh, PA 15261, USA; jonnysax@gmail.com (J.F.); mis23@pitt.edu (M.S.); donna.stolz@pitt.edu (D.B.S.); 3 Wilmer Eye Institute, The Johns Hopkins University School of Medicine, Baltimore, MD 21287, USA; szigler45@gmail.com

**Keywords:** retina pigment epithelium, explant culture, RPE flatmount, adenoviral transduction

## Abstract

Degeneration of retinal pigment epithelium (RPE) is one of the most critical phenotypic changes of age-related macular degeneration (AMD), the leading cause of vision loss in the elderly. While cultured polarized RPE cells with original properties are valuable in *in vitro* models to study RPE biology and the consequences of genetic and/or pharmacological manipulations, the procedure to establish mouse primary PRE cell culture or pluripotent stem cell-derived RPE cells is time-consuming and yields a limited number of cells. Thus, establishing a mouse *in situ* RPE culture system is highly desirable. Here we describe a novel and efficient method for RPE explant culture that allows for obtaining biologically relevant RPE cells *in situ*. These RPE explants (herein referred to as RPE flatmounts) are viable in culture for at least 7 days, can be efficiently transduced with adenoviral constructs, and/or treated with a variety of drugs/chemicals followed by downstream analysis of the signaling pathways/biological processes of interest, such as assessment of the autophagy flux, inflammatory response, and receptor tyrosine kinases stimulation. This method of RPE explant culture is highly beneficial for pharmacological and mechanistic studies in the field of RPE biology and AMD research.

## 1. Introduction

The retinal pigment epithelium (RPE) is a monolayer of highly polarized cells that performs multiple functions to support retinal health, such as phagocytosis of photoreceptor outer segments, autophagy, transporting nutrients and materials for the visual cycle, maintaining the blood–retinal barrier, etc. [1]. RPE degeneration has been identified as a key feature in many retinal diseases, for instance, retinitis pigmentosa, Stargardt disease, and age-related macular degeneration (AMD) [2]. Thus, understanding RPE cell biology and metabolism is imperative for retina researchers.

A number of cell models exist to study RPE biology. RPE cell lines from rodents and humans that have been transformed (RPE-J, hTERT-RPE1) or spontaneously immortalized (ARPE-19) are widely used due to the simplicity of their maintenance *in vitro,* similar to most other cell lines [3]. However, these flat and non-pigmented cells are not fully differentiated; they do not resemble RPE cells *in vivo* and generally have much lower expression or do not express signature RPE proteins that are essential to the native RPE to maintain RPE homeostasis [1]. Hazim et al. reported a rapid way to induce differentiation of APRE19 cells with nicotinamide, which resulted in establishment of cultures with improved RPE properties such as cell polarity, expression of signature RPE genes, and phagocytosis. However, the limitations of this technique are low transepithelial resistance, lack of pigmentation, and abnormal karyotype [4].

Another option is obtaining RPE cells from mouse- or human-induced pluripotent stem cells (iPS-RPE) or embryonic stem cells (ES-RPE) by culturing them in differentiating medium either in dishes or on semi-permeable transwell filters to allow fluid flow and to enhance polarization. These cells possess many RPE-specific characteristics and the procedure usually yields more cells compared to primary RPE cultures [5]. However, obtaining well-differentiated RPE cells from either iPS cells or ES cells is expensive and time-consuming (typically 6–12 weeks) [6,7], which may diminish enthusiasm for working with them.

To overcome these difficulties, primary RPE cells isolated and cultured from rodents, pigs, or human eyes are commonly used in many RPE studies. The main benefit of this method is that the cells maintain most of the original properties, such as RPE-specific protein expression, pigmentation, polarity, etc. [8,9,10]. However, the availability of human tissues for RPE cultures is very limited. RPE primary cell cultures from pigs are not common either due to the cost. In contrast, mouse models are used the most in the biomedical field, including vision research. Thus, primary RPE cells from mice are highly sought after. However, the small and intricate structure of mouse eyeballs limits the amount of mouse RPE cells that can be isolated and thus a primary cell culture has been regarded as a technically challenging and time-consuming procedure. In some studies, freshly dissected RPE/Choroid/Sclera tissue explants were collected for immediate live cell imaging; however, these explants were viable *in vitro* for only a couple of hours, which significantly limited their scope of application [11,12].

In this study, we established a new method to culture viable RPE explants that can be used for various downstream manipulations and analyses, including but not limited to the measurements of autophagy flux, inflammatory response, and receptor tyrosine kinases stimulation. We also describe the use of adenovirus vectors to efficiently express genes of interest in RPE flatmounts *in vitro*, which improves gain- and loss-of-function studies as well as investigations on signaling pathways compared to any other previously described methods.

## 2. Results

### 2.1. RPE Flatmounts Are Viable after 7 Days in Culture

**Figure 1:**
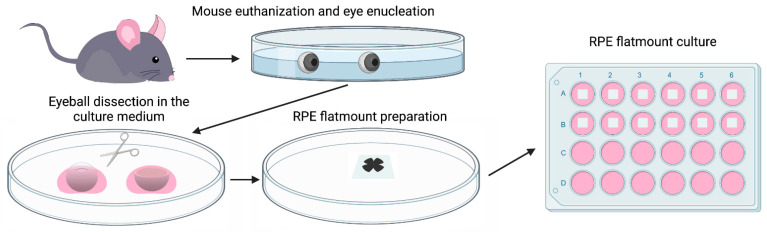
Diagram showing the procedure of establishing a mouse RPE explant culture. Eyes were enucleated from a euthanized wild-type C57BL/6J mouse, transferred to sterile PBS, and washed with PBS twice. After removing the connective tissue and anterior parts (cornea and lens), the resulting eyecups were cut into four petals. Neural retinae were then peeled off and the RPE/Choroid/Sclera complexes were mounted on a PVDF membrane with the sclera attached to the membrane. RPE flatmounts were then transferred to a 24 well plate with a 500 μL culture medium (with the RPE facing down) and cultured in an incubator at 37 °C with 5% CO_2_.

Wild-type C57BL/6J mice were used to prepare RPE flatmounts in this study. Briefly, the mice were sacrificed by CO_2_ asphyxiation, and the eyes were enucleated and transferred immediately to PBS for washing. Posterior eyecups were obtained after careful removal of the cornea, lens, and iris pigment epithelium. After the eyecups were cut into four petals and the neural retinas removed, RPE/Choroid/Sclera complexes were mounted on a PVDF membrane with the sclera attached to the membrane. RPE flatmounts were then transferred to a 24 well plate with a culture medium (Figure 1). The detailed culture method is described in the Materials and Methods section.

**Figure 2:**
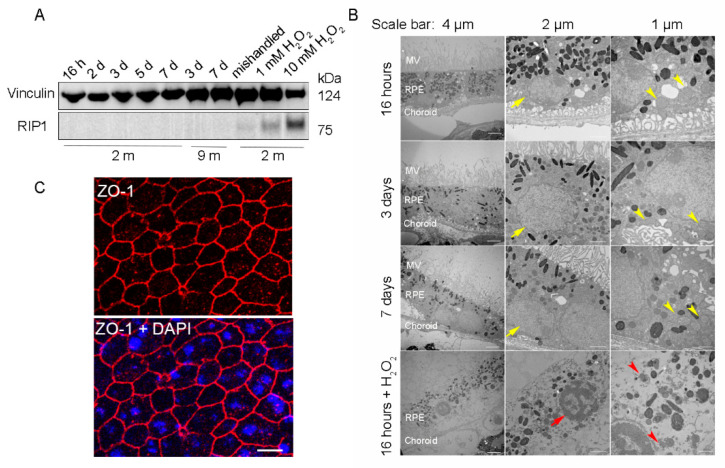
RPE flatmounts are viable for at least one week after culture *in vitro*. (**A**) RPE flatmounts were prepared from 2- and 9-month-old wild-type C57BL/6J mice and cultured in complete medium with or without H_2_O_2_ for up to one week. Protein lysates were prepared from the RPE flatmounts and subjected to Western blotting analysis. The cell death marker RIP1 was used to evaluate cell viability; vinculin was used as an internal control. As a positive control: RPE flatmounts treated with either 1 mM or 10 mM H_2_O_2_ had significant expression of RIP1. Further, the RPE flatmount that was cultured for 7 days without changing the medium (mishandled sample) showed a slight amount of RIP1, whereas the RPE flatmounts cultured with frequent medium changes did not express RIP1, suggesting that the RPE cells obtained even from older mice (9 months) were viable after at least 7 days of culture. (**B**) RPE flatmounts were obtained from 3-month-old wild-type C57BL/6J mice and cultured in complete medium with or without H_2_O_2_ treatment for up to one week. RPE flatmounts were then fixed in 2.5% glutaraldehyde and processed for transmission electron microscopy (TEM). The TEM imaging of the RPE flatmounts cultured for 16 h, 3 days, and 7 days showed that these RPE cells have normal nuclei (yellow arrows) and organelles, such as mitochondria (yellow arrowheads), and abundant microvilli. H_2_O_2_-treated RPE flatmounts showed cell death symptoms, including chromatin condensation and fragmentation (red arrow), a disrupted cell membrane, complete loss of microvilli, a translucent cytoplasm, and severely damaged mitochondria (red arrowheads). (**C**) ZO-1 immunostaining on 2-month-old RPE flatmounts cultured for 3 days showed robust ZO-1 expression, indicating RPE cell integrity.

To ensure that the RPE cells were viable for further downstream experiments, RPE flatmounts were cultured for up to 7 days and analyzed with western blotting and transmission electron microscopy (TEM) to evaluate the cell death and ultrastructural changes, respectively. RPE flatmounts treated with 1 mM or 10 mM hydrogen peroxide (H_2_O_2_) for 16 h served as a positive control for cell death [13]. RPE flatmounts cultured for 7 days without replacing the medium every other day (“mishandled” sample) was used as a potential additional positive control for cell death. Cell death was assayed by detecting the protein levels of Receptor-Interacting Protein Kinase 1 (RIP1), which is a positive regulator of apoptosis and necrosis in response to cell death stimuli [14]. RIP1 was significantly expressed in H_2_O_2_ (either 10 mM or 1 mM)-treated RPE flatmounts; the RPE flatmounts without fresh medium replacement also showed a trace of RIP1 expression (Figure 2A). On the contrary, RPE flatmounts cultured normally from either 2- or 9-month-old mice showed no RIP1 expression (Figure 2A), indicating that the RPE cells were viable even after 7 days of culture (Figure 2A). TEM was also performed to evaluate the health of the RPE in flatmounts obtained from 3-month-old mice. The RPE cells treated with 10 mM H_2_O_2_ had severe ultrastructural changes, such as microvilli loss, thinner cytoplasm with severely damaged mitochondria (red arrow heads, Figure 2B), nuclear fragmentation (red arrows, Figure 2B), and loss of membrane integrity. In contrast, the TEM images from the RPE flatmounts cultured normally for 16 h, 3 days, and 7 days revealed abundant and healthy microvilli, healthy mitochondria with normal cristae (yellow arrow heads, Figure 2B), and well-shaped nuclei (yellow arrows, Figure 2B), further confirming the viability of these flatmounts. We also performed ZO-1 immunostaining on RPE flatmounts cultured for 3 days. The data (Figure 2C) indicated a robust expression of ZO-1 in RPE flatmounts and therefore confirmed the RPE integrity.

### 2.2. RPE Explants Abundantly Express RPE Signature Genes for 3 Days in Culture

**Figure 3:**
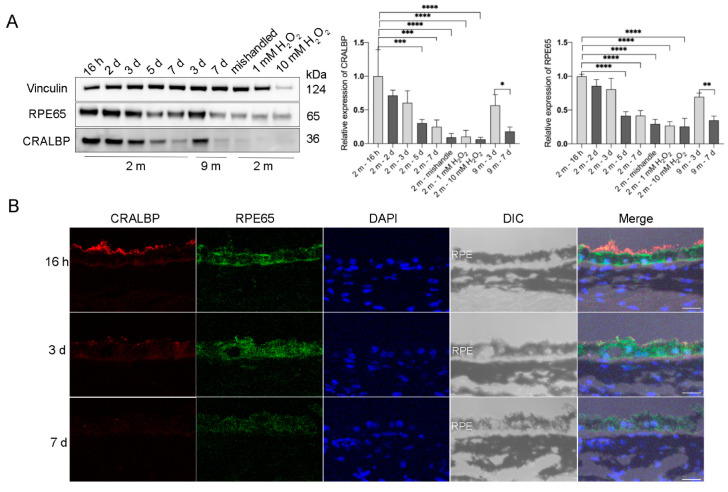
Visual cycle proteins were abundantly expressed in the RPE flatmounts during the first 3 days in culture, but gradually decreased over time. RPE flatmounts obtained from 2- and 9-month-old wild-type C57BL/6J mice were cultured in complete medium for up to 7 days. RPE flatmounts treated with H_2_O_2_ were cultured for 16 h. (**A**) Protein lysates were prepared from the RPE flatmounts and subjected to western blotting analysis to detect the levels of visual cycle proteins RPE65 and CRALBP. Statistical analysis was performed using one-way ANOVA. * *p* < 0.05, ** *p* < 0.01, *** *p* < 0.001, **** *p* < 0.0001. n = 3. (**B**) RPE65 and CRALBP protein levels were also measured by immunofluorescence. Data from both the western blotting and immunofluorescence showed the gradual reduction in the two proteins in RPE flatmounts over time. Scale bar = 20 μm.

To test if the cultured RPE explants retain the original properties of RPE cells *in vivo*, we measured the protein expression of the RPE signature proteins that are involved in the visual cycle: Retinal Pigment Epithelium-Specific Protein 65 kDa (RPE65) [15] and cellular retinaldehyde-binding protein (CRALBP) [16]. Both RPE65 and CRALBP were abundantly expressed in RPE flatmounts within 3 days of culture and there was no significant difference in the expression levels during these first 3 days. However, the expression of these two proteins started to decrease after 3 days of culture. After the 7th day, the expression of RPE65 and CRALBP was still detectable, but markedly decreased compared to the level observed during the first 3 days (Figure 3A). Notably, RPE cells treated with H_2_O_2_ for 16 h or RPE flatmounts left in culture for 7 days without fresh medium replacement both had significantly decreased RPE65 and CRALBP expression (Figure 3A). The immunofluorescence staining for RPE65 and CRALBP performed on RPE flatmount sections also confirmed the western blot results (Figure 3B).

### 2.3. Adenoviral Vector Is a Highly Efficient Tool for Genetic Manipulations in RPE Flatmounts

**Figure 4:**
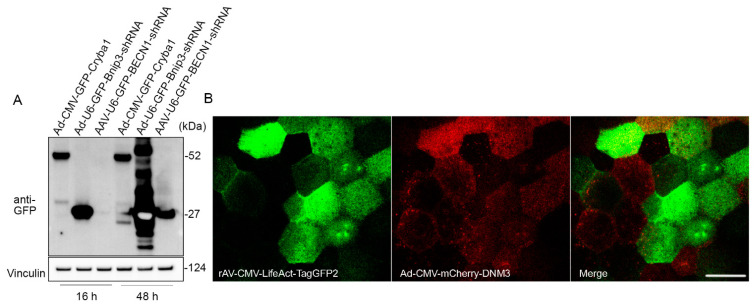
Adenoviral vector is an efficient tool for gene delivery in RPE flatmounts. (**A**) RPE flatmounts were infected with either adenoviral (Ad) or adeno-associated viral (AAV) constructs. All these constructs were tagged with GFP either fused or not fused with a protein. RPE flatmounts were infected either with the Ad constructs at a concentration of 8 × 10^7^ PFU/mL or with AAV constructs at a concentration of 4.5 × 10^9^ GC/mL. Fluorescent tag GFPs were driven by CMV promoters in both the overexpression vector and shRNA vector. The infected RPE flatmounts were collected either after 16 h or 48 h of incubation with the viral constructs. Western blotting data of the GFP levels suggested a much higher infection efficiency with adenovirus compared to AAV2, suggesting that RPE flatmounts are readily susceptible to adenovirus infection. (**B**) RPE flatmounts were infected with rAV-CMV-LifeAct-TagGFP2 and Ad-CMV-mCherry-DNM3 for 16 h. RPE flatmounts were then subjected to imaging using confocal microscopy. Scale bar = 20 μm.

Primary RPE cells and IPS-derived RPE cells are known for their extremely low plasmid transfection efficiency [1]. In this study, we tested the transduction efficiency of adenoviral and adeno-associated viral vectors in flatmount preparations. RPE flatmounts from 2-month-old wild-type C57BL/6J mice were infected either with the adenovirus constructs at a concentration of 8 × 10^7^ PFU/mL or with adeno-associated viral (AAV) at a concentration of 4.5 × 10^9^ GC/mL. All of the viral constructs had a GFP tag driven by a CMV promoter (for both overexpression and shRNA vectors). RPE flatmounts were collected after 16 h or 48 h of incubation with the viral constructs. Western blotting analysis showed a significant overexpression of GFP in RPE flatmounts transduced by either overexpression or shRNA adenoviral virus after overnight incubation (16 h), but not in those transduced by the AAV vectors (Figure 4A). The GFP expression level was much higher after 48 h of incubation with AAV vectors compared to 16 h incubation; however, the AAV vectors still had a lower transduction efficiency compared to the adenoviral vectors (Figure 4A).

If spatial localization of the transgene is desired, the RPE flatmounts can either be imaged using an engineered dish lid as described previously [17] or fixed for immunostaining. Figure 4B shows an example of confocal imaging on a live RPE flatmount transduced with Ad-CMV-mCherry-DNM3 (expressing mCherry-fused Dynamin 3) and rAV-CMV-LifeAct-TagGFP2 (expressing a LifeAct-GFP fusion protein to fluorescently label the F-actin cytoskeleton).

### 2.4. RPE Explant Cultures Can Be Used for Various Treatments and Downstream Analyses

We next tested whether the cultured RPE flatmounts could be used to introduce various treatments required for mechanistic studies. We have evaluated three different biological events in RPE flatmounts. For all three experiments, RPE flatmounts were prepared from wild-type C57BL/6J mice and cultured overnight (16–18 h) before treatment.

**Figure 5:**
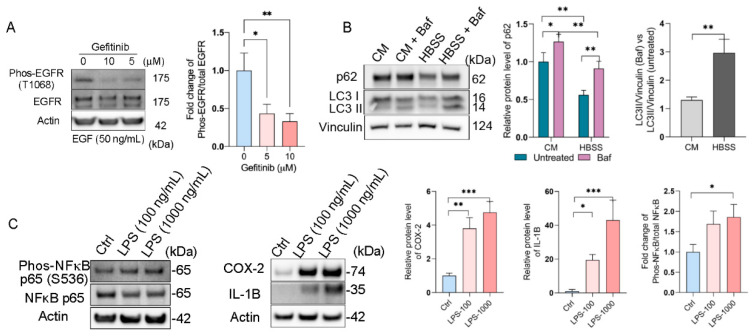
RPE flatmount culture is valid for downstream treatments. (**A**) RPE flatmounts obtained from 3-month-old C57BL/6J wild-type mice were starved in DMEM only medium and incubated with Gefitinib (EGFR inhibitor) for 3 h. RPE flatmounts were then stimulated with EGF for 30 min. Data showed the inhibition of EGFR activation by Gefitinib. (**B**) Autophagy flux measurements in RPE flatmounts with or without starvation. RPE flatmounts were obtained from 8-month-old C57BL/6J wild-type mice and cultured in complete medium for 16 h, and then either cultured in complete medium with Bafilomycin A1 or starved in HBSS with Bafilomycin A1 for 3 h. RPE flatmounts were then collected for western blotting analysis. The data showed an increase in LC3II accumulation (after the blockage of autolysosome formation by Bafilomycin A1) and a decrease in p62 expression in the starved RPE flatmounts, suggesting enhanced autophagy flux after starvation. (**C**) RPE flatmounts obtained from 8-month-old C57BL/6J wild-type mice and cultured in complete medium with or without different doses of LPS for 24 h were collected and analyzed for an inflammatory response. The COX-2, IL-1B, and phosphorylated NF-κB protein levels were measured by western blotting and the data showed significant upregulation of the respective proteins in LPS-treated RPE flatmounts. Statistical analysis was performed using one-way ANOVA. * *p* < 0.05, ** *p* < 0.01, *** *p* < 0.001. *n* = 3.

First, we evaluated the epidermal growth factor receptor (EGFR) response upon epidermal growth factor (EGF) stimulation on RPE flatmounts. We previously stimulated RPE flatmounts with EGF in a time-dependent manner and determined the activation of EGFR. Western blot analysis showed that phosphorylated EGFR was significantly increased after 5 min of EGF stimulation and remained high through 60 min of stimulation, and the total EGFR was decreased over time due to degradation, as described [17], indicating the RPE flatmounts in culture were responsive to extracellular stimuli such as EGF, and very likely other growth factors. In this study, we also treated the RPE flatmounts with the EGFR inhibitor, Gefitinib. The western blot analysis showed an inhibition of EGFR activation by Gefitinib (Figure 5A), indicating the RPE flatmount culture system can be used for drug studies.

An autophagy flux assay was also performed on the RPE flatmounts (Figure 5B). It is known that starvation induces autophagy flux [18]. RPE flatmounts were starved in Hank’s balanced salt solution (HBSS) with or without Bafilomycin A1, a specific vacuolar H  +  ATPase (V-ATPase) inhibitor that blocks the fusion between autophagosomes and lysosomes [19]. RPE flatmounts were collected after 3 h of incubation and subjected to western blotting analysis. Microtubule-associated protein light chain 3 (LC3) is involved in autophagosome formation and now is widely used to monitor autophagy. The common approach is to detect the conversion of LC3I to LC3II, which is correlated with the number of autophagosomes [20]. The western blotting data clearly showed more accumulation of LC3II in the starved RPE flatmount with Bafilomycin A1 compared to that in the RPE flatmounts with complete medium and Bafilomycin A1, indicating increased autophagosome formation and enhanced autophagy flux caused by starvation (Figure 5B). Additionally, we tested the levels of p62/SQSTM1 protein, a ubiquitin-binding scaffold protein that colocalizes with ubiquitinated protein aggregates, and thus is widely used as a marker to study autophagic flux. p62/SQSTM1 accumulates when autophagy is inhibited, and its decreased levels can be observed when autophagy is induced [21]. In starved RPE flatmounts, the p62/SQSTM1 protein level was much lower compared to that of the fed RPE flatmounts with or without Bafilomycin A1 (Figure 5B).

We also evaluated the inflammatory response in the RPE flatmounts using lipopolysaccharide (LPS). Western blots showed significantly increased expression of phosphorylated NFκB, COX-2, and IL-1B in LPS-treated RPE cells in a dose-dependent fashion (Figure 5C). NFκB, IL-1B, and COX-2 are all important mediators of inflammatory pathways and are activated in response to various extracellular or intracellular physiological stimuli, including LPS administration [22,23,24]. These data suggest a significantly activated inflammatory response in RPE flatmounts treated with LPS.

Taken together, our data demonstrate that a cultured RPE flatmount is a suitable *in vitro* model to study the diverse molecular mechanisms in the RPE and the pathobiology of retinal diseases such as AMD.

## 3. Discussion

*In vitro* RPE models provide valuable tools for studying RPE cell biology and its implication in AMD. The method for culturing RPE explants that we have established and described here provides a quick and efficient *in vitro* system for performing molecular biology and biochemistry studies, including, but not limited to, protein analysis by western blotting, ELISA, immunostaining, TEM, quantitative PCR, etc. This method can be applied to both wild-type mice or genetically engineered mice [17]. We have successfully cultured RPE flatmounts obtained from mice ranging from 2 weeks to 9 months of age; however, we speculate that RPE flatmounts obtained from older mice can also be successfully cultured.

Some limitations of this method are worth stating. Although these RPE cells *in situ* are highly competent in maintaining their original properties, we found that the protein levels of some RPE signature genes, visual cycle genes in particular, were decreased in the explants after 3 days in culture. This may be attributed to the loss of interaction with photoreceptors. In addition, dissecting proficiency is needed for the RPE flatmount preparation. The RPE cells should be disturbed as little as possible during the dissection process and flatmount preparation.

Finally, we found that the polarized and tightly connected RPE monolayers are very susceptible to transduction using adenoviral constructs. It is known that RPE primary cells and stem cell-derived RPE monolayers are resistant to plasmid transfection, thus reducing the selection of tools for introducing transgenes into the RPE. Based on our experience, adenoviral vectors should be exploited over traditional transfection to introduce the transgenes since the transduction efficiency is extremely high for both the RPE cells *in situ* as well as the pluripotent stem cell-derived RPE monolayers.

Taken together, considering the good viability and susceptibility to adenoviral transductions and drug treatments, RPE explants might be a better alternative to the less biologically relevant RPE cell lines or to the more technically challenging primary RPE cultures. RPE explants might be useful tools for researchers who work on understanding the pathogenesis of AMD—a prevalent multifactorial eye disease among the elderly population that can lead to blindness. There is currently no effective therapy for dry AMD. While many factors are known to contribute to AMD pathogenesis, extensive studies have shown that RPE degeneration plays a leading role in the onset and progression of this disease. RPE degeneration can ensue when oxidative stress, inflammation, lysosomal clearance, and epigenetics are dysregulated in RPE cells [25,26,27,28].

Even with some concerns about the absence of macula in the mouse retina, mouse models are still widely used in AMD research, due to the similar RPE and rod/cone density as the human perifovea, high genetic manipulability, short life span, and low maintenance cost. We recommend using this novel method of culturing mouse RPE explants to study RPE biology, including various aspects of RPE contribution to AMD pathogenesis.

## 4. Materials and Methods

### 4.1. Animals

All C57BL/6J mice used in this study were purchased from the Jackson Laboratory (Connecticut, USA). All animal studies were conducted in accordance with the Guide for the Care and Use of Animals (National Academy Press) and were approved by the Animal Care and Use Committee of the University of Pittsburgh (approved 20 October 2020-present).

### 4.2. RPE Explant Culture

#### 4.2.1. Preparation

RPE explant culture medium was made using Dulbecco’s Modified Eagle’s Medium (DMEM, 4.5 g/L glucose, Gibco) with 2% Fetal Bovine Serum (Sigma Aldrich, St. Louis, MO, USA), 1% Penicillin–Streptomycin (Gibco), 1% GlutaMAX™ Supplement (100×, Gibco), and 1% MEM Non-Essential Amino Acids Solution (100×, Gibco, Amarillo, TX, USA). The medium was pre-warmed in a 37 °C incubator before use. The dissecting instruments, sterilized with 70% ethanol, as well as sterile phosphate-buffered saline (PBS, 1×, pH 7.4, Gibco) and 60 mm and 100 mm sterile Petri dishes (Nunc) were placed in a laminar flow cabinet.

#### 4.2.2. Obtaining Mouse Eyes and Dissection

The mouse was euthanized by CO_2_ asphyxiation followed by cervical dislocation and placed on an absorbent pad. Ethanol (70%) was sprayed around the eye area. The tip of angled scissors (Noyes Scissors, 12 cm, Curved, 15 mm blades, WPI) was inserted between the skin and the eyeball, carefully cutting out the eye and placing it immediately in sterile PBS on a 60 mm Petri dish. The Petri dish with eyes was moved into the laminar flow cabinet for further processing. The eyes were washed one more time with sterile PBS in a fresh 60 mm Petri dish. Next, the eyeballs were transferred to another 60 mm Petri dish containing a warm culture medium. From this, one eye was placed in a 100 mm Petri dish under a stereomicroscope (Leica, Wetzlar, Germany) placed in the laminar flow cabinet. Toothed forceps (Castroviejo suturing forceps 0.12 mm teeth, WPI, Worcester, MA, USA) and Vannas scissors (Vannas Capsulotomy Scissors, Curved, WPI) were used to carefully remove the connective tissue around the eyeball, taking care not to cut into the sclera. After clearing away the connective tissue, the eye was placed in a drop of the culture medium. The eye was carefully held in position with forceps and either an incision was made around the ora serrata with Vannas scissors or simply a tiny hole was made using a needle prick under the dissecting microscope. Keeping the eyeball in the culture medium, the anterior cornea was carefully removed by extending the cut around the ora serrata circumference and pulling the anterior cornea away after the cut was complete. Following this, the lens and associated iris pigmented epithelium were removed by gently pulling them out of the eyecup with toothed forceps. The iris epithelium and ciliary body should be removed so that the neural retina can be easily peeled off from the RPE layer in Step 3. The resulting posterior eyecup was then transferred to a fresh drop of culture medium. The eyecup was then cut into four petals under the dissecting microscope, such that the cuts were long enough to flatten the eyecup but short enough to keep the petals connected.

#### 4.2.3. RPE Flatmount Preparation

One of the following two ways was used to flatten the RPE/Choroid/Sclera complex on a supporting membrane: (1) In a drop of culture medium, the neural retina was carefully removed by gently pulling it from the edges with forceps while holding the remaining RPE/Choroid/Sclera complex steady with another pair of forceps. In a fresh drop of culture medium, the RPE/Choroid/Sclera complex was flipped with the RPE side facing the dish bottom. The drop was then delicately aspirated to flatten the 4 petals. Next, a 6 × 6 mm PVDF membrane (22860, Thermo Fisher Scientific, Waltham, MA, USA) was placed on top of the sclera. The PVDF membrane with the attached RPE/Choroid/Sclera complex was then flipped back and the petals were neatly and gently spaced out on the membrane using forceps if necessary. (2) The eyecup (pre-cut into 4 petals) was placed on a 6 × 6 mm PVDF membrane and flattened, followed by gently pulling away the neural retina from the edges with one pair of forceps while holding the remaining RPE/Choroid/Sclera complex steady with another pair of forceps. The dissection should be performed gently, disturbing the RPE cells as little as possible.

#### 4.2.4. RPE Flatmount Culture

Each RPE flatmount was placed in a well of a 24-well plate containing 500 μL of the culture medium. Since the flatmount on the membrane floats in the culture medium, it is necessary to keep the RPE cells facing the bottom of the dish. The plate was then placed in a 37 °C incubator with 5% CO_2_. The same technique was used to process the next eye. The prepared RPE explants were then used for genetic manipulation using adenovirus constructs and/or pharmacological manipulation. For long-term culture, the medium was replaced with fresh medium every other day by gentle aspiration of the existing medium and adding fresh medium without removing the membrane with the RPE from the well.

### 4.3. RPE Flatmount Treatments

For all treatments reported in this study, the RPE flatmounts were cultured in complete medium overnight (16–18 h) before treatment. For the EGF stimulation assay, RPE flatmounts obtained from 3-month-old C57BL/6J were starved in DMEM-only medium and incubated with 5 μM and 10 μM of Gefitinib for 3 h. RPE flatmounts were then stimulated with 50 ng/μL EGF (AF-100-15, Peprotech, Cranbury, NJ, USA) for 30 min. For the lipopolysaccharides (LPS) treatment assay, RPE flatmounts obtained from 8-month-old C57BL/6J mice were treated with 100 ng/mL and 1 μg/mL LPS for 24 h in culture. For the autophagy flux assay, RPE flatmounts obtained from 8-month-old C57BL/6J mice were either cultured in complete medium or starved in HBSS for 3 h. Bafilomycin A1 was used to block autolysosome formation, and the accumulation of LC3II was measured to evaluate the autophagy flux. Following the treatments, the RPE flatmounts attached to the membrane were washed with PBS, the PVDF membrane was removed by gentle pulling the RPE from the membrane using forceps, and the RPE flatmounts were snap-frozen on dry ice.

### 4.4. Protein Isolation and Western Blotting

The snap-frozen RPE/Choroid/Sclera complexes were thawed to proceed with protein isolation. RPE cells were released into RIPA buffer from the RPE/Choroid/Sclera complexes by tapping and vortexing for 30 s in RIPA lysis buffer (20-188, Millipore, Burlington, MA, USA) supplemented with a protease and phosphatase inhibitor cocktail as described [17]. Choroid/Sclera complexes were then removed from the lysate buffer using forceps. The RPE cells in RIPA buffer were sonicated for 15 s and incubated on ice for 15 min, followed by centrifugation at 15,000 rpm for 20 min to clear the protein lysates from the cell debris. The protein concentrations were determined by BCA assay (23225, Thermo Fisher Scientific). The native protein samples were mixed with 4× protein LDS sample buffer (NP0007, Invitrogen, Waltham, MA, USA) containing 10% 2-mercaptoethanol and denatured at 100 °C for 10 min. In total, 8~12 mg of protein from each RPE sample were loaded onto a 4–12% Bis-Tris Nu-PAGE gel (NP0323BOX, Invitrogen) and transferred to a nitrocellulose membrane. Blots were blocked in either 5% non-fat milk (170-6404, BioRad, Hercules, CA, USA) or 5% BSA (A3912-50G, Sigma Aldrich), and then incubated with primary antibodies overnight at 4 °C. After being washed 3 times, the blots were incubated in the appropriate HRP-conjugated secondary antibody for 1 h at room temperature. Blots were visualized using the ECL Western blotting detection reagent (RPN2209, GE Healthcare Life Sciences, Singapore). Antibodies were used in this study: Phos-EGFR (Tyr1068) (1:1000, #2234, Cell Signaling Technology, Danvers, MA, USA), EGFR (1:1000, #2646, Cell Signaling Technology), Phos-NFκB p65 (S536) (1:1000, #3033, Cell Signaling Technology), NFκB p65 (1:1000, #8242, Cell Signaling Technology), COX2 (1:1000, #12282, Cell Signaling Technology), IL-1B (1:1000, ab9722, Abcam, Cambridge, UK), LC3B (1:1000, #2775, Cell Signaling Technology), p62(1:1000, NBP1-48320, Novos Biologicals, Littleton, CO, USA), RPE65 (1:1000, MA5-32633, Invitrogen), CRALBP (1:1000, MA1813, Thermo Fisher Scientific), RIP1 (1:1000, MAB3585, R & D Systems, Minneapolis, MN, USA), Actin (1:1000, A2066, Sigma Aldrich), and Vinculin (1:1000, ab73412, Abcam).

### 4.5. RPE Flatmount Sectioning and Immunostaining

RPE flatmount cryosections were permeabilized with 0.25% Triton X-100 in PBS for 10 min and incubated in blocking buffer (2% donkey serum, 2% Goat serum, 1% BSA, and 0.1% Triton X-100 in PBS) for 30 min at room temperature followed by incubation in RPE65 antibody (1:200, MA5-32633, Invitrogen) and CRALBP antibody (1:200, MA1813, Thermo Fisher Scientific) in blocking buffer overnight at 4 °C. Sections were then washed with 1× PBS and incubated with the secondary antibody (1:200, Goat anti-Rabbit, Alexa Fluor 488, A21206, Invitrogen; 1:200, Donkey anti-Mouse IgG (H + L), Alexa Fluor 555, A-31570, Invitrogen) and 1μg/mL DAPI (D1306, Thermo Fisher) in blocking buffer for 1 h at room temperature. After washing, sections were mounted with DAKO mounting medium (S3023, Agilent) and sealed with a coverslip. For the ZO-1 immunostaining on RPE flatmounts, whole RPE flatmounts were permeabilized with 0.25% Triton X-100 in PBS for 10 min, and incubated in blocking buffer (5% BSA, and 0.1% Triton X-100 in PBS) for 30 min at room temperature followed by incubation in ZO-1 antibody, Alexa Fluor 594, (1:200, 339194, Invitrogen) in blocking buffer overnight at 4 °C. Flatmounts were then washed with 1× PBS and incubated with 1μg/mL DAPI in PBS for 10 min at room temperature. After washing, flatmounts were flattened on a microscope slide with the RPE layer up. The flatmounts were then mounted with DAKO mounting medium and sealed with a coverslip. Images were acquired on a Zeiss LSM 710 confocal workstation.

### 4.6. Transmission Electron Microscopy

RPE flatmounts (with the PVDF membrane) were fixed in 2.5% glutaraldehyde (Energy Beam Sciences, East Granby, CT, USA) in 1× PBS (pH 7.4) for 2 h at room temperature and cut to the proper size. The specimens were then washed 3 times with 1× PBS (15 min each wash) and postfixed in 1% osmium tetroxide (Osmium Tetroxide crystals, Electron Microscopy Sciences, Hatfield, PA, USA) with 1% potassium ferricyanide (Fisher Scientific, Pittsburgh, PA, USA) overnight followed by 3 washes in 1x PBS for 15 min each. Samples were then dehydrated in a graded series of ethanol (30%, 50%, 70% in PBS, 90% in H_2_O_2_, 100% × 3) for 15 min each (30–95%) in reagent alcohol (Fisher Scientific) and 100% requisitioned in pints and then in two 10-min incubations in propylene oxide (Electron Microscopy Sciences). The tissues were infiltrated with a 1:1 mix of propylene oxide and epon (Energy Beam Sciences) overnight followed by incubation with pure epon overnight at 4° C, and further infiltrated with three 1-h changes of epon. Samples were finally embedded in pure epon at 37 °C for 24 h and cured for 48 h at 60 °C. Ultra-thin sections were cut with a Leica EM UC7 Ultramicrotome (Leica, Wetzlar, Germany), stained with uranyl acetate (Electron Microscopy Sciences) and lead citrate (Fisher Scientific), and visualized with a JEM-1400Flash Electron Microscope (JEOL, Tokyo, Japan).

### 4.7. Viral Vector Transduction and Imaging on RPE Flatmounts

The Ad-CMV-GFP-*Cryba1*, Ad-CMV-mCherry-*Dnm3*, Ad-U6-GFP-*Bnip3*-shRNA, and AAV2-U6-GFP-*Becn1*-shRNA vectors were made by Vector Biolabs (Pittsburgh, PA, USA). Both Ad-CMV-GFP-*Cryba1* and Ad-CMV-mCherry-*Dnm3* are CMV promoter-driven vectors that contain mouse *Cryba1* cDNA (NM_009965) fused with GFP tag and mouse *Dnm3* cDNA (BC141144) fused with mCherry tag, respectively. Both Ad-U6-GFP-Bnip3-shRNA and AAV2-U6-GFP-BECN1-shRNA have separate CMV promoters for GFP. rAV-CMV-LifeAct-TagGFP2 is an adenoviral construct and was purchased from ibidi (60121), containing a LifeAct-GFP fusion protein to fluorescently label the F-actin cytoskeleton. RPE flatmounts obtained from 2-month-old C57BL/6J mice were infected with Ad-CMV-GFP-Cryba1, Ad-U6-GFP-Bnip3-shRNA, and AAV2-U6-GFP-BECN1-shRNA vectors at a concentration of 8 × 10^7^ PFU/mL (Ad constructs) and 4.5 × 10^9^ GC/mL (AAV2 constructs). RPE flatmounts were collected after 16 h or 48 h of incubation for western blotting analysis. RPE flatmounts obtained from 3-month-old C57BL/6J mice were cultured in glass-bottom dishes and co-infected with rAV-CMV-LifeAct-TagGFP2 and Ad-CMV-mCherry-DNM3 at the concentration of 4.4 × 10^7^ PFU/mL and 5 × 10^6^ IU/mL, respectively, for 16 h. As described previously [17], the imaging dish was clipped with a 3D-printed black lid, and the RPE flatmount was compressed with a plunger, axially positioned using a fine pitch thread such that the apical surface of the RPE cells was close enough to the glass bottom, and the RPE cells could be imaged.

### 4.8. Statistical Analysis

Statistical analysis was performed using GraphPad Prism v.8 software. All graphs show the mean ± S.D. The *p*-values were obtained by either one-way ANOVA (multiple comparisons) or a two-tailed unpaired Student’s *t*-test (comparison between two groups). The number of biological repeats (n) of each experiment are provided in the figure legends.
